# Cardiac Hypoxia Tolerance in Fish: From Functional Responses to Cell Signals

**DOI:** 10.3390/ijms24021460

**Published:** 2023-01-11

**Authors:** Maria Carmela Cerra, Mariacristina Filice, Alessia Caferro, Rosa Mazza, Alfonsina Gattuso, Sandra Imbrogno

**Affiliations:** Department of Biology, Ecology and Earth Sciences, University of Calabria, 87036 Arcavacata di Rende, Italy

**Keywords:** fish heart, cyprinids, contractility, metabolism, nitric oxide

## Abstract

Aquatic animals are increasingly challenged by O_2_ fluctuations as a result of global warming, as well as eutrophication processes. Teleost fish show important species-specific adaptability to O_2_ deprivation, moving from intolerance to a full tolerance of hypoxia and even anoxia. An example is provided by members of *Cyprinidae* which includes species that are amongst the most tolerant hypoxia/anoxia teleosts. Living at low water O_2_ requires the mandatory preservation of the cardiac function to support the metabolic and hemodynamic requirements of organ and tissues which sustain whole organism performance. A number of orchestrated events, from metabolism to behavior, converge to shape the heart response to the restricted availability of the gas, also limiting the potential damages for cells and tissues. In cyprinids, the heart is extraordinarily able to activate peculiar strategies of functional preservation. Accordingly, by using these teleosts as models of tolerance to low O_2_, we will synthesize and discuss literature data to describe the functional changes, and the major molecular events that allow the heart of these fish to sustain adaptability to O_2_ deprivation. By crossing the boundaries of basic research and environmental physiology, this information may be of interest also in a translational perspective, and in the context of conservative physiology, in which the output of the research is applicable to environmental management and decision making.

## 1. Introduction

Oxygen fluctuations are a common experience for species living in water environments, since they naturally occur over a diurnal/seasonal rate and are exacerbated by eventual anthropic manipulation that challenge the O_2_ budget. Exposure to natural O_2_ variations potently shaped the evolution of a number of adaptive strategies that, in fish, require behavioral, morphological and functional modifications. Reaching the surface to breathe the uppermost layer of water in contact with air, increasing the activity to avoid the hypoxic area, or decreasing the activity to reduce O_2_ demand, are amongst the most common behavioral responses to low O_2_ [1,2]. Beyond them, changes in ventilation and hemoglobin–O_2_ binding [3] contribute to ameliorate O_2_ extraction from the environment in order to maintain aerobic ATP production. 

Fundamental for fish adaptation to restricted O_2_ is a proper availability of metabolic fuels, obtained either by reducing energy consumption or increasing substrates extraction from energy stores, or both. This is accompanied by an appropriate blood supply for cells and tissues, provided by the compensatory adaptation of both the heart and the circulatory system. All the above responses are supported by cellular and molecular adaptive rearrangements that contribute to an orchestrated framework of events allowing to preserve body functions, while at the same time protecting from the risk of metabolic impairment. 

At the extremes of the large spectrum of adaptation to O_2_ availability, several fish species evolved the ability to survive even in the presence of O_2_ below the critical tension (Pcrit), thus tolerating prolonged hypoxia and/or anoxia [3,4,5]. An example is represented by the teleost belonging to cyprinids, which are champions of hypoxia/anoxia tolerance. For this peculiarity, there is a continuously growing interest to analyze the physiological mechanisms that, at a different degree of biological organization, make cyprinid species able to cope with restrictions in O_2_.

In this review, we aim to summarize recent and classic literature to show the strategic role of the heart in the adaptive ability of cyprinids to hypoxia/anoxia. The contribution of metabolic reorganization for optimizing energy availability and protecting from waste accumulation, as well as the fundamental role of the nitrergic system as a major player in the cardiac functional response to hypoxia, will be illustrated. For the unfamiliar reader, information will be also provided on the adaptive performance of the fish heart to the challenge of reduced O_2_. In this regard, data will be analyzed by taking into account the major frame of reference represented by the rich literature on hypoxia-intolerant species, highlighting, when available, data on cyprinids and other hypoxia-tolerant fish.

## 2. The Challenge of Hypoxia for the Pumping Fish Heart

Hypoxia imposes conflicting demands on cardio-respiratory function. Being systemically O_2_ supply-dependent on cardiac output (CO) and arterial O_2_ concentration, fish can respond to and cope with hypoxia through cardio-respiratory adjustments to preserve systemic O_2_ delivery, thus maintaining aerobic metabolism, or by reducing O_2_ demands via anaerobic metabolism or metabolic depression [3,6,7]. Depending on the time of hypoxia exposure (i.e., acute or chronic), the heart may undergo different stimulation, and thus, may activate different responses. Accordingly, the effects of low O_2_ for the pumping fish heart require consideration by taking into account whether O_2_ deprivation occurs acutely or is accompanied by a long-term adaptation.

### 2.1. Acute Hypoxia

Hyperventilation, stimulated by chemoreceptors sensitive to water and/or blood PO_2_ concentrations, represents a physiological mechanism by which fish attempt to maintain O_2_ consumption to face a rapidly declining environmental O_2_. Consequent activation of chemoreceptors activates an immediate response that contributes to O_2_ uptake regulation thanks to a reflex increase in gill ventilation frequency and/or amplitude [8,9]. Along with ventilatory adjustments, elevated levels of circulating catecholamines may also occur that confer protection during hypoxia [10].

At the cardiac level, exposure to hypoxia is accompanied by a reflex bradycardia, mediated by vagal inhibition [11] and by increased systemic resistance [12]. This is considered a protective strategy when O_2_ supply is low. It ameliorates both electrical and mechanical cardiac activity, which is crucial to preserve performance during hypoxia [11]. A lower heart rate (HR) is associated with a prolonged cardiac action potential [13,14] and increased systolic calcium transients. This is consistent with the negative force-frequency relationship (i.e., contractile force decreases as contraction frequency rises) typical of the fish heart [15] that allows for the reduction of diastolic calcium levels, thus increasing systolic calcium transients [15]. An increased diastolic interval also favors the residence time of blood in the lumen of the heart (i.e., more time for O_2_ diffusion), improving myocardial oxygen extraction. Moreover, an increased stroke volume (SV) by stretching the cardiac chambers may reduce O_2_ diffusion distances. Further benefits of hypoxic bradycardia include a reduced O_2_ demand, obtained by depressing the power output, and an increased coronary blood flow, due to a prolonged diastole. This enhances O_2_ delivery to the heart in species with coronary circulation [11,16]. Different from this general picture, hypoxia bradycardia is absent in lungfish that extract O_2_ from the air, in Antarctic teleosts, and in hypoxia-tolerant species (for ref. see [11]). It is also lacking in early embryonic and larval stages when a cholinergic control is not yet established. In the zebrafish *Danio rerio*, it first appeared in juvenile fish (30 days post fertilization) [17,18], while tachycardia is present in zebrafish larvae (4 days post fertilization) [19] when the heart is sensitive to adrenergic, but not cholinergic, stimulation [20,21]. This is intriguing since zebrafish change O_2_ sensitivity during development, moving from hypoxia tolerance to intolerance during growth [17]. Also in the hypoxia-sensitive trout, adrenergic tonus is established early in development [22] and is able to mediate tachycardia until the maturation of vagal control allows for the “switch” from hypoxic tachycardia to bradycardia [23].

During acute hypoxia CO remains constant or slightly increases in species such as rainbow trout *Oncorhynchus mykiss*, Atlantic cod *Gadus morhua* and Atlantic hagfish *Myxine glutinosa* [16,24,25] due to increased venous pressure and ventricular filling time, which enhance stroke volume [11,16]. In fact, contrary to mammals, fish enhance cardiac output mainly via larger changes in stroke volume than in heart rate [26,27]. Interestingly, a normal or enhanced cardiac function is present in species showing hypoxia/anoxia tolerance, including several cyprinids. In the common carp *Cyprinus carpio* and in its related specie, the crucian carp *Carassius carassius*, a strong metabolic depression (about 30%) is fundamental to survive anoxia, although interspecific differences have emerged. In fact, while in the common carp the cardiac function is strongly depressed during 24 h of severe hypoxia, the crucian carp conserved normal cardiac activity and autonomic cardiovascular control in up to 5 days of anoxia at 8◦C [28]. Two different strategies are engaged by these species to face reduced oxygen availability: in the anoxia-tolerant crucian carp (*C. carassius*), the cardiac Power Output (PO), i.e., the product of cardiac output and ventral aortic blood pressure (an index of cardiac ATP demand) [29] is routinely kept below the maximal glycolytic capacity, even under normoxic conditions, thus avoiding the need to reduce it during hypoxia [28]; in contrast, in the hypoxia-tolerant common carp (*C. carpio*), hypoxic bradycardia decreases cardiac PO in order to reduce cardiac ATP demand within a level that can be supported by glycolytic ATP production [29]. These responses indicate that a depressed cardiac PO may represent a key component of hypoxia tolerance, allowing to match cardiac energy demand with reduced energy supply. A reduced cardiac ATP demand via bradycardia has also been reported in the hypoxia-tolerant tilapia [30]. The hemodynamic analysis of the ex vivo isolated working heart of the goldfish *Carassius auratus* acutely exposed to hypoxia showed a time-dependent increase in stroke volume, indicative of a potentiated performance [31]. This was particularly evident under preload increases (i.e., the Frank–Starling response) in which the maximum SV was reached at input pressures lower than the normoxic heart. This feature, which appears a prerogative of the goldfish, is proposed as a mechanism to properly support organ perfusion, thus preventing tissue intoxication [31].

### 2.2. Chronic Hypoxia

Although the cardiac effects of chronic hypoxia received limited attention, the few available data on hypoxia-intolerant species show that the response differs depending on species, time, activity, and degree of exposure. An example is the Atlantic cod, in which hypoxic acclimation saw an unchanged HR at rest [25,32], but an increase under high swimming speeds. Differently, a decreased in vivo cardiac SV and CO is documented both at rest and during swimming [32]. Similar results have been obtained in steelhead trout *Oncorhynchus mykiss* exposed to chronic moderate hypoxia [33], suggesting a hypoxia-dependent impaired myocardial contractile performance following chronic exposure to hypoxia. Of note, the inability to raise cardiac output is accompanied by an improved tissue O_2_ extraction for steelhead trout and Atlantic cod [32,33] thus compensating for diminished cardiac pumping capacity. In isolated ventricular trabeculae from hypoxia-acclimated rainbow trout, the shortening work and power (indicative of the ability to eject blood from the heart), but not the lengthening work, were significantly reduced [34]. On the basis of these results, authors suggested that the decreased SV documented in trout and cod exposed to chronic hypoxia [32,33] results from an increased end-systolic volume (i.e., a decrease in ejection fraction).

In the channel catfish, a hypoxia-tolerant species, moderate hypoxia-acclimation significantly increased heart rate [35,36]. In zebrafish, the ability to respond to acute hypoxia (after the stage of 30 days) appears more effective in the animals raised under chronic hypoxic exposition, suggesting that acclimation to moderate hypoxic conditions improves their tolerance to acute environmental hypoxia [17]. In the crucian carp (*C. carassius*), conceivably the most anoxia-tolerant fish species, a dependence of anoxia tolerance on the temperature has been earlier demonstrated by Blazka [37]; furthermore, its anoxia tolerance varies seasonally, as indicated by a better anoxia tolerance in the winter-acclimatized fish in comparison to carp caught in summer [38]. Recently, it was observed that crucian carp acclimated at typical winter temperature responds to anoxia with a sustained bradycardia, the results of which are energetically beneficial [39]. As proposed, while hypoxic bradycardia allows more time for oxygen transfer from water to blood and then to cardiac myocytes [11], anoxic bradycardia may represent an advantage by reducing energy consumption, thus improving survival of the heart under prolonged seasonal anoxia [39]. At the same time, cold-acclimated crucian carp shows a remarkable lengthening of ventricular action potential (AP) duration. This allows for a constant diastole/systole duration which is important for ensuring tissue perfusion at low heart rate under anoxia [39]. Of note, while in mammalian hearts hypoxia results in an accelerated ventricular AP occurring via the opening of the ATP-sensitive K^+^ channels [40], in the crucian carp these channels are not activated under prolonged anoxia [41]. This is different from the slight shortening of ventricular AP observed in the heart of warm-acclimated goldfish in which exposure to hypoxia is associated with the opening of the ATP-sensitive K^+^ channels [42]. It remains a question whether the dissimilar behavior shown by the crucian carp and the goldfish is a peculiar species-specific trait or if it depends on different experimental temperatures and oxygen regimes.

## 3. Hypoxia-Related Metabolic Responses of the Fish Heart

In 1986, Hochachka firstly proposed metabolic arrest, i.e., a simultaneous reduction in metabolic rate and metabolic demands, as a key adaptation to O_2_ deprivation in organisms capable of long-term anoxic survival [43]. Contrary to the activation of the anaerobic pathway to sustain ATP production and maintain aerobic respiratory rates, the reduction in energetic demand, which clues to an overall reduction in ATP turnover, preserves glycogen stores and avoids the accumulation of waste products (i.e., acid lactic production) which may rapidly lead to a Pasteur effect. Thus, organisms tolerating long-term anoxia lack a Pasteur effect [44]. In fact, they do not increase glycolytic ATP production to maintain aerobic respiratory rates. This general concept has endured over time and is corroborated by new findings related to the identification of alternative metabolic pathways which allow a switch to anaerobic metabolism keeping low waste product accumulation. The extreme is exemplified by the capacity of cyprinid fish to tolerate prolonged O_2_ absence by using large glycogen stores to generate ethanol as a by-product of energy metabolism, thus avoiding acidosis [45]. This extraordinary capacity is due to the presence in Carassius genus (*C. carassius* and *C. auratus*) of an alternative E1 pyruvate dehydrogenase enzyme, one of the catalytic components of the pyruvate dehydrogenase complex (PDHC) which, under anoxia, functions as an acetaldehyde-producing mitochondrial pyruvate decarboxylase (PDC) analogous to the cytosolic pyruvate decarboxylase in brewer’s yeast [46]. This isoform derives from an additional set of paralogs for each of the E1α and E1β sub-units, originating from a cyprinid-specific paleotetraploidization event occurring approximately 8.2 million years ago in a common ancestor of the Carassius genus (anoxia tolerant) and the common carp (anoxia intolerant) [46]. While one pair maintained the original function (i.e., catalyzing the synthesis of acetyl-CoA during normoxia as an integral part of PDHC), the other pair has apparently evolved into an E1 enzyme physically independent of PDHC, catalyzing the formation of acetaldehyde in anoxia, which then can be effectively converted into ethanol by a muscle-specific alcohol dehydrogenase (ADH). Authors [46] reported a tissue-specific distribution of PDHC sub-units with E1α3, E1β2, and E2a transcripts dominating in ethanol-producing red and white skeletal muscle, and E1α1 or E1α2, E1β1, and E2b transcripts in heart, brain, and liver, with expression levels lower than muscle; this suggests a minor role for these tissues in ethanol production under anoxia. Nonetheless, the heart of these species conserve normal cardiac activity if exposed to acute hypoxia (*C. auratus*: [31]), or up to 5 days of anoxia (*C. carassius*: [28]). A preserved heart performance is the basis for improving anoxia resistance of the whole piscine organism since it ensures metabolic and functional cooperation among single organs [47]. In this view, an appropriate perfusion of organ and tissues guarantees lactate transport to the muscle for its conversion into ethanol and, the latter, to the gills for excretion.

**Figure 1:**
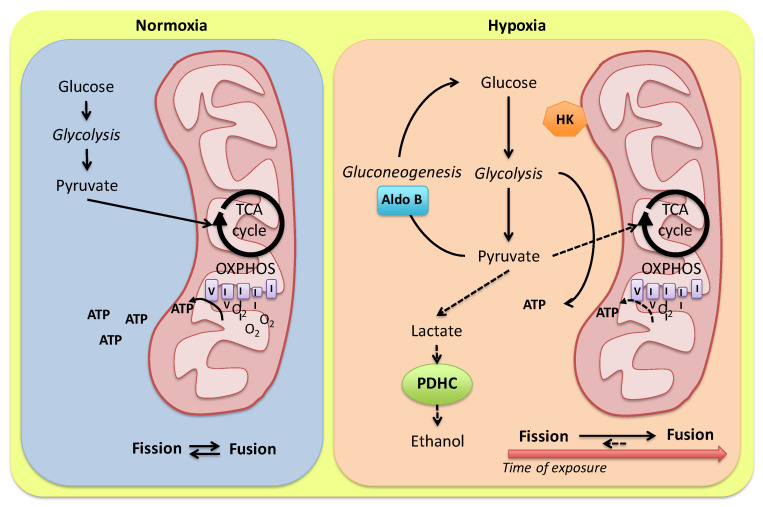
Model for alternative routes of pyruvate metabolism in the *Carassius auratus* heart. In the presence of O_2_, pyruvate from glycolysis is converted to acetyl-CoA and addressed to mitochondria for the oxidative phosphorylation. Under hypoxia, a tight modulation of glycolysis enzymes may finely regulate anaerobic ATP production by modulating glycolytic vs. gluconeogenic flux. Pyruvate conversion to ethanol is reduced by a low cardiac expression of alternative PDHC sub-units (see the text for details). A hypoxia-dependent modulation of mitochondria dynamics in relation to the exposure time is proposed. HK: hexokinase; Aldo B: aldolase B; PDHC: pyruvate dehydrogenase complex.

The Carassius ability to maintain routine cardiac activity during anoxia implies a cardiac ATP demand which is lower than their maximum glycolytic potential [29], thus protecting the heart from the accumulation of anaerobic waste products. In the goldfish, the enhancement of myocardial contractility in response to low O_2_ is associated with low amounts of cardiac lactate together with a slight reduction in pyruvate levels [48]. By using mass spectrometry-based proteomic analysis, authors identified two isoforms of fructose-bisphosphate aldolase, i.e., aldolase C and aldolase B, differently expressed in homogenates of goldfish heart exposed to normoxic or hypoxic medium, with aldolase B predominantly expressed in the hypoxic heart [48]. Aldolase catalyzes the reversible conversion of fructose-1,6-bisphosphate to glyceraldehyde 3-phosphate (G3P) and dihydroxyacetone phosphate (DHAP). While aldolase C appears to be more effective in participating in glycolysis, aldolase B has evolved to have a role in gluconeogenesis [49,50]. This supports the possibility that in the goldfish exposed to reduced O_2_, a tight modulation of the aldolase enzyme isoforms may finely regulate glycolytic vs. gluconeogenic flux, thus enhancing anaerobic ATP yield and minimizing metabolic acidosis [48]. Of note, under O_2_ limitation, a number of glycolytic enzymes show increased binding to subcellular components, particularly mitochondria [51] or the particulate fraction, and this is proposed to finely regulate glycolytic flux rates through the modulation of enzyme-specific kinetics [52] (Figure 1). Examples are represented by the increased binding of hexokinase to mitochondria observed in the heart of goldfish maintained in anoxic water [53], as well as the increased binding capacity of phosphofructokinase, aldolase, and pyruvate kinase to the particulate fraction observed in ventricular sheets of armored catfish (*Liposarcus pardalis*) exposed to hypoxia [52].

The cardiac response of fish to low O_2_ requires an analysis in relation to fuel substrates. Carbohydrates represent the energy source of choice for the heart of several fish species [54]. In the isolated and perfused eel heart, glucose supply maintains the cardiac performance during acute anoxia (see references in [55]) while, in the American eel *Anguilla rostrata*, characterized by a marked anoxic endurance, the inhibition of oxidative phosphorylation with NaCN activates glycogen stores degradation regardless of glucose levels in the medium [56]. Prolonged survival under anoxia requires large stores of fermentable substrate (normally glycogen), whose conservation is facilitated by a strong metabolic depression [44]. In the anoxic crucian carp, cardiac glycogen stores are quickly mobilized during the first week of anoxia with little further degradation when anoxia is protracted to 3 and 6 weeks [57]. This suggests that, after the first week of anoxia, the heart performance relies on exogenous glucose. Of note, glycogen depletion is not paralleled by increased glucose or lactate concentration, the latter even reduced if compared to the normoxic control [57]. It has been proposed that the early mobilization of glycogen (and other glycolytic intermediates) is not an advantage to the fish in relation to its anoxia tolerance, but it is crucial for adapting body fluids osmolarity, which is perturbed in the presence of an increase in body mass (6.2%) occurring under anoxia [57]. An accumulation of glycolytic intermediates has also been detected in the cardiac and skeletal muscle of anoxic goldfish [58]. Protracted hypoxia (2.1 kPa for 4 weeks) suppresses goldfish metabolic rate by 74% [59] in the whole animal with no direct effects on the heart, which retains a normal mitochondrial respiration rate [60]; carbohydrates represent the election fuel in maintaining mitochondrial respiration [60]. This apparently contrasts with in vitro data showing that, on ventricular strips from goldfish, hypoxia depresses myocardial contractility and O_2_ consumption rate [61]. However, this does not significantly change O_2_ utilization capacity (i.e., the ratio of twitch force to O_2_ consumption), and this may reflect a lower activation of anaerobic energy production. This suggests that the goldfish heart is able to maintain a higher degree of aerobic metabolism at low O_2_ tensions without increasing anaerobic energy production [61].

Because of their ability to detect changes in O_2_ availability, mitochondria are known for their role in coordinating the responses to low O_2_ [62]. Mitochondrial respiration is differently affected by hypoxia acclimation depending on metabolic fuels, species, and tissue. In fish, many studies on the effects on mitochondrial function under hypoxia and/or anoxia-reoxygenation have been mainly performed on muscle and liver tissues [63,64,65]. Only a few investigations examined the effects of chronic hypoxia on cardiac mitochondrial function, providing conflicting information. By using permeabilized cardiac fibers and isolated mitochondria, Cook et al. [66] showed no effect on complex I and II respiration in juvenile snapper (*Pagrus auratus*) acclimated to 10.2–12.1 kPa for 6 weeks. In contrast, oxidative phosphorylation decreased in permeabilized ventricle fibers from the hypoxia-intolerant shovelnose ray (*Aptychotrema rostrata*) following a 2 h in vivo hypoxic insult, while it was preserved in the hypoxia-tolerant epaulette shark (*Hemiscyllum ocellatum*) [67]. In the hypoxia-tolerant sablefish (*Anoplopoma fimbria Pallas*), cardiac mitochondrial respiration was maintained following exposure to chronic hypoxia (8 ± 1 kPa for 6 months) and similar P50 values between normoxic and hypoxic groups suggest that the O_2_ dependence of complex IV, the primary site of O_2_ consumption in the mitochondrion, is not affected by low O_2_ acclimation [68]. In addition, the cardiac activity of citrate synthase, a marker of oxidative capacity, increases in both ventricular homogenates and mitochondrial suspension of hypoxia-acclimated sablefish [68], suggesting that a sustained cardiac mitochondrial capacity primarily involves changes in the intrinsic properties of the mitochondria and not in the abundance of these organelles [69]. In the goldfish, chronic hypoxia decreased COX activity in different tissues, except for the heart [60].

An increased activity of the oxidative phosphorylation enzymes often coincides with an enhancement of the percentage of cell volume displaced by mitochondria [70]. Different stimuli may affect the mitochondrial compartment in fish. Examples are the increased mitochondrial density documented in response to cold acclimation in the oxidative muscle fibers of European eel (*Anguilla anguilla*), striped bass (*Morone saxatilis*), crucian carp (*C. carassius*), goldfish (*C. auratus*) and stickleback (*Gasterosteus aculeatus*) [70], as well as in the hypertrophic zebrafish heart in response to humoral stimulation by angiotensin II [71], and in the ventricle of the European eel during ontogenetic growth [72]. Of note, in the hypoxia-acclimated goldfish heart, a modulation of transcripts coding for mitochondrial fission (*fis1*) and fusion (*mfn1* and *mfn2*) proteins has been reported [73]. Specifically, 1-week exposure to hypoxia elicits a significant reduction in the relative transcript abundance of mitofusin *mfn1* and an increase in the mitochondrial fission factor *fis1* [73], calling for activation of the fission process. On the contrary, protracting hypoxia to 4 weeks, the relative transcript abundance of mitochondrial fusion and fission proteins is restored at normoxic values [73]. This modulation of mitochondria dynamics allows for the hypothesis that in the early phase of hypoxia adaptation, an augmented mitochondrial density, due to the activation of fission events, may maximize energy delivery to the contractile apparatus needed to sustain the enhanced pumping behavior of the heart [31]. In contrast, if hypoxia is protracted, mitochondrial fusion occurs and promotes mitochondrial membrane stability to protect mitochondria from damage, mitophagy and the induction of cellular apoptosis [73] (Figure 1).

## 4. The NOS/NO System as a Cardiac Molecular Actor in the Hypoxia Response

Experimental evidence of the last decade has recognized the crucial role of the gasotransmitter NO and its derivatives in the molecular mechanisms that sustain heart function under hypoxia (e.g., [74,75,76,77,78]). NO is mainly produced by NO synthases (NOSs) isoenzymes (i.e., the constitutive endothelial (eNOS; NOS3) and neuronal (nNOS; NOS1), and the inducible (iNOS; NOS2) isoforms) which catalyze the oxidation of the guanidino group of L-arginine with molecular O_2_ to produce L-citrulline and NO. The stringent dependance by O_2_ makes the NOS enzyme susceptible to a hypoxia-related modulation. To date, *nos* genes have been found in teleosts, with the exception of *nos3*, whose identification so far remains elusive, despite several approaches that suggest the presence of all NOS enzymes in fish (see for references [47,79,80]). In fact, by using physio-pharmacological approaches, NADPH-diaphorase and immunolocalization studies with mammalian anti-eNOS antibodies, an endocardial-endothelial NO source involved in cardiac modulation was demonstrated in several teleost species [55,81,82,83,84]. In fish, NOS1-type proteins appear more closely related to NOS3 than to NOS2 proteins [75]. It has been proposed that some functional traits of the eNOS isoform are covered by a (set of) nNOS isoform(s), showing an endothelial-like consensus [85]. Accordingly, it is possible that one of the different isoforms evolved to provide the cell with eNOS-like functions [75].

In oxygenated media, NO is rapidly metabolized to nitrite (NO_2_^−^) and nitrate (NO_3_^−^). Since the reactions leading to NO_3_^−^ production are slower than those leading to NO_2_^−^ [86], NO_2_^−^ is considered the major NO metabolite. Nitrite and nitrate represent a bioavailable reservoir of NO in blood and tissues [87,88]. The reduction of nitrite to NO may occur via acidic disproportionation [89], or enzymatic reduction via xanthine oxidoreductase, mitochondrial enzymes or deoxygenated Hb, Mb, cytoglobin-1, neuroglobin, globin-X and eNOS (see references in [90,91,92,93]). Nitrate may also contribute to NO homeostasis, since it can be reduced to nitrite by xanthine oxidoreductase [94,95]. However, under hypoxic conditions, the production of NO from nitrite seems to be more pronounced. Thus, depending on O_2_ tension, a balance between the oxidative pathway (NOS) and the reductive pathway (NO_2_^−^) of NO production preserves NO homeostasis. Of note, O_2_ levels can impact the oxidation/reduction properties of heme- and molybdopterin-containing proteins, so that proteins that at physiological O_2_ conditions are involved in oxidative processes can become reductive enzymes able to catalyze the reduction of nitro compounds to release NO when O_2_ is lacking [96,97,98].

During hypoxia or anoxia, when NOS enzymes are unable to produce NO, the possibility to maintain internal nitrite levels is particularly important for securing NO availability. If compared to terrestrial animals, in fish, an important source of nitrite for the internal NO generation is represented by the exogenous supply. It has been reported that when exposed to deep hypoxia, the crucian carp takes up ambient nitrite across the gills and directs it to tissues, including the heart [99]. Of note, the carp, as other hypoxia-tolerant species, such as the goldfish, show basal plasma nitrite levels (0.75–1.75 µM) higher than those reported in hypoxia-intolerant fish (e.g., European flounder *Platichthys flesus*, eelpout *Zoarces viviparus*, oyster toadfish *Opsanus tau*, brown trout *Salmo trutta*) (about 0.2 µM) [100,101,102]. As shown in the zebrafish, exposure to high nitrite is accompanied by high levels of HbNO, a biomarker of NO generation from nitrate [103]. However, at high concentrations nitrite is toxic and can influence ion, respiratory and circulatory homeostasis [101]. Moreover, a high nitrite-derived NO could perturb physiological processes, and may induce tissue nitrosative stress, resulting in high levels of S-nitrosylated proteins and cell damage [101]. For these reasons, fish living in nitrite-contaminated environments need to balance the advantages of a rich ambient pool of nitrite for internal NO production with the potentially dangerous effects of nitrite-polluted habitats [104].

NO has numerous potential reactions that may influence a variety of physiological and pathophysiological processes. The direct interaction of NO with metal-containing proteins or with organic free radicals represents two of the best characterized direct effects of NO in biological systems. The reaction of NO with certain metals to form nitrosyl complexes occurs in vivo primarily with iron-containing proteins [105]. A well-known reaction of NO is with proteins that contain a heme moiety to form stable nitrosyl adducts. The most notable of these is the interaction of NO with guanylate cyclase, which leads to the formation of cGMP [106,107,108,109]. cGMP has several regulatory effects, including modulation of the vascular tone, angiogenesis and vascular remodeling, and inhibition of platelet aggregation [110,111]. Yet, this same type of chemistry can also inhibit other metalloproteins such as cytochrome P-450, NOS, cytochrome oxidase and catalase [105].

In addition to being a signal transduction agent through reversible reactions with heme protein targets, the radical character of NO makes it a central player in free radical and redox biology. NO shows a limited chemical reactivity and, consequently, its direct toxicity is less than that of reactive O_2_ species (ROS). However, it is able to react with O_2_^−^, producing peroxynitrite anion (ONOO^−^) [112], a very damaging species [113]. Peroxynitrite may lead to the formation of secondary oxidizing species (i.e., hydroxyl radicals (OH^•^), carbonate radicals (CO_3_^•−^) and nitrogen dioxide (NO_2_^•^)), that cause oxidative modifications of biomolecules, including thiol oxidation and tyrosine nitration, thus causing permanent modifications of cellular components and severe alterations of cell and mitochondrial homeostasis [114]. By reacting with molecular O_2_ and nitrogen, nitric oxide forms nitrogen dioxide or dinitrogen trioxide, both toxic oxidizing and nitrosating agents [112]. Collectively, nitric oxide, nitrogen dioxide and peroxynitrite represent reactive nitrogen species (RNS) capable of damaging lipids, proteins and DNA [114].

In fish, NO is an important signaling molecule involved in many physiological processes. Currently, a still growing body of literature is available on its role in the modulation of the fish heart [55,82,115,116,117,118]. Data on trout alevins (*Salmo trutta*) show that L-arginine-derived NO reduces heart rate, while NOS inhibition induces tachycardia, an event that was considered an indirect consequence of vasoconstriction [119]. However, a direct chronotropic control mediated by the gas is reported in developing zebrafish, in which NOS inhibition was found to depress heart rate, and also to induce arrhythmic behavior [120]. Interestingly, in fish NO is involved also in shaping early cardiac development. It is reported in the zebrafish that administration of an exogenous NO donor (DEANO) induces a change in heart position, the organ being located to the right side, instead of the left side of the embryo. This situ inversion was proposed to occur by controlling cardiac progenitor cell migration [121]. Of note, despite the lack of direct evidence, it is possible that the above NO-dependent events are under an O_2_-dependent modulation. In fact, it is known that limited hypoxia is beneficial for zebrafish cardiac development [122].

In adult fish, NO is deeply involved in controlling cardiac performance under basal and stressful conditions [31,82,83,115,116,123]. Many of the observed effects have been related to the specific source of the gas. For example, in ventricular strips from trout and goldfish, the NOS-derived NO inhibits respiration rate and improves myocardial efficiency [61], while in trout but not in goldfish, the NO generated from nitrite conversion reduces O_2_ consumption without changing force development. Species-specific differences in O_2_ affinity of cardiac Mb and then, in its nitrite reductase capacity, have been used to explain these different responses. In fact, under conditions of low O_2_, Mb may readily de-oxygenate and generate NO from nitrite in trout, while in the goldfish, Mb remains saturated with O_2_, and thus prevents nitrite reduction [61].

As shown in mammals, during O_2_ limitation, NO influences mitochondrial signaling [124] and modulates mitochondrial O_2_ consumption and ROS production [125,126,127,128]. These effects are mainly related to NO’s competition for O_2_ binding sites on cytochrome oxidase (complex IV) [129,130,131] and the S-nitrosylation of complex I [132,133,134]. In the goldfish heart, NO inhibits mitochondrial respiration without changing contractility [61]; in the presence of reduced O_2_, this sustains myocardial function, thus contributing to myocardial efficiency [28]. This is in agreement with the enhanced performance shown by the heart of the goldfish when exposed to acute hypoxia, and is correlated to an increased NO production [31]. Of note, in the goldfish heart, the hypoxia-induced increase in NO levels could activate sarcolemmal KATP channels, a response that may enhance hypoxia tolerance [135], similarly to the mammalian preconditioning protection of ischemic myocardium, in which the opening of ATP-sensitive K+ channels represent a crucial event [136]. Interestingly, the potentiated heart function observed in the goldfish heart exposed to hypoxia is accompanied by an increased expression of NOS, which likely helps to keep adequate myocardial NO levels [31]. The major role of NO in the increase in contractility observed in the goldfish heart exposed to hypoxia is supported by data obtained when the hypoxic heart is treated with the NO scavenger PTIO, as well as with the NOS inhibitor L-NMMA [31]. Moreover, an activation of the PI3-K/Akt signaling has been observed in the hypoxic goldfish heart [76], evidence that clearly resembles the molecular pattern that in mammals controls NO generation through eNOS activation [137,138].

Of note, in the goldfish heart exposed to hypoxia, the increased NOS expression is accompanied by an enhanced expression of HIF1α, suggesting a role in the NO/HIF1a system in the cardiac response to decreased O_2_ [31]. In fish, HIF1a is expressed in the heart of several species, such as the Atlantic croaker (*Micropogonias undulatus*; [139]) and the Antarctic red-blooded teleost *Notothenia coriiceps* [140], and is positively modulated by hypoxia, an effect which is reversed by the restoration of normoxic O_2_ values (for a recent review see, e.g., [141]). The parallel enhancement of HIFα and NOS expression observed in the goldfish heart under O_2_ limitation is noticeable since it is similar to the events occurring in the ischemic mammalian myocardium, in which HIF-1α contributes to cell survival by activating hypoxia-related genes, including *Nos* [142,143,144,145]. It has been reported in mammals that at high concentrations (>1 μM) NO may stabilize HIF-1α that, after dimerization, binds HIF responsive elements, thus promoting NOS expression [146]. Although specific evidence on a putative interplay between HIF-1α and NOS is still lacking in the fish myocardium, the available information suggests that the relationship between these important molecular mediators represents a crucial pathway of the cardiac response to hypoxia. It also suggests that this pathway appeared early in the evolution and is retained up through the vertebrates.

The intracellular targets activated by NO have been widely assessed in fish, particularly in relation to its role as a major organizer of complex cardiac transduction signals [55,82,83,147,148]. More recent studies pointed the attention to the molecular targets involved in the control of the cardiac response to low O_2_. In this regard, it has been proposed that under hypoxic conditions in fish the cardiac downstream NO activated pathways do not involve cGMP-activation [76]. cGMP-independent pathways recently emerged as an important route for NO to control its molecular targets. In particular, the degree of protein S-nitrosylation, the covalent attachment of NO to the thiol group of cysteine (Cys) residues, significantly decreases in the hypoxic goldfish heart with respect to the normoxic counterpart [76]. In mammals, dysregulated protein S-nitrosylation has been correlated with either cardiac disorders [149] or with the activation of protective mechanisms against the development of stress-induced myocardial dysfunction [150]. Although information about the type of proteins encountering denitrosylation is not yet available in the hypoxic fish heart, it is possible that, under hypoxic conditions, this process may activate protective programs, thus contributing to preserving the myocardium [76]. In addition, in the hypoxic goldfish heart, the reduction of protein S-nitrosylation is accompanied by an increased expression of Nox2, the catalytic sub-unit of NADPH oxidase [76], and of 3-nitrotyrosine [151]. This suggests that NO may modulate the response of the fish heart to hypoxia by utilizing protein nitration, i.e., the substitution, mainly under the action of peroxynitrite (ONOO^–^), of a nitro group to tyrosine residues. The nitration process has been generally associated with alterations of protein catalysis, protein–protein interaction, and tyrosine kinase signaling [152]; however, a nitration-dependent control of redox homeostasis has also been observed in normally functioning cardiac muscle [153].

**Figure 2:**
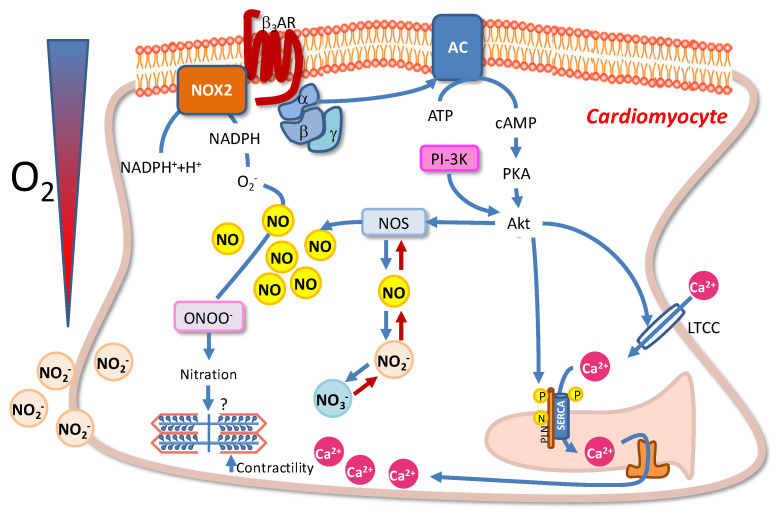
Schematic overview of the NOS/NO-mediated intracellular pathways activated in goldfish cardiomyocytes under hypoxic stress. For details, see the text.

The presence of cysteine and tyrosine residues makes several proteins possible targets of nitrosative and oxidative modifications [153,154]. Amongst others, the SERCA2a pump, the protein controlling the calcium-dependent homeostatic myocardiocytes activity [155], is of particular interest to understand the events occurring in the goldfish heart under hypoxia. Its structural proximity to mitochondria exposes it to reactive O_2_/nitrogen species generated as by-products of the oxidative phosphorylation [156]. Of note, nitrated SERCA2a is used as a cardiac marker of nitrative stress [153]. The inhibition of the SERCA2a pump, which is expressed in the fish heart [157,158], is accompanied by a significant reduction of the hypoxia-induced increase in the goldfish heart performance [76]. This is in line with possible involvement of SERCA2a pump in the nitrergic-dependent control of the response of the fish heart to low O_2_. These data open the possibility that, in fish, NO may activate a protective program that contributes to sustaining the performance of the heart challenged by hypoxia. In light of the NO-dependent modulation of the cardiac sarcolemmal KATP channels observed in the goldfish heart [135], this response, similarly to the KATP-dependent protection observed in the ischemic mammalian myocardium [136], may contribute to the cardiac hypoxia tolerance of this teleost. In this perspective, a relationship between NO and other cardioprotective substances may be hypothesized. For example, it has been recently observed that in the goldfish hypoxia induces an increase in cardiac β3-adrenoceptors-(ARs) expression [159], and that the pretreatment of the isolated working heart with a selective β3-ARs inhibitor (the SR59230A) abolishes the hypoxia-dependent increase in myocardial contractility [159]. The cardioprotective role of β3-ARs is well documented in mammals [160]. In addition, in mammals, the β3-AR is upregulated in response to hypoxia, and by activating the NO signaling, it is involved in the angiogenic responses to hypoxia [161]. Although yet to be elucidated, the above evidence suggests that in hypoxia-tolerant fish (e.g., the goldfish), NO may coordinate the complex networks triggered by humoral cardioprotective mediators. An overview of the role of nitric oxide and its metabolites in the modulation of the goldfish heart performance is depicted in Figure 2.

## 5. Conclusions

A growing effort of research significantly contributed in the last decades to uncovering a number of strategies that, from genes to the whole organ, allow the heart of several teleost species to cope with hypoxia, enabling adaptation and survival under conditions mostly detrimental for non-adaptable species. Although many gaps are still present, the evidence of aspects of unity in the cardiac response to low oxygen in terms of whole heart functional responses, metabolic reorganization, and the fundamental role of molecular systems, such as the NOS/NO pathway and its related signals, makes the information available so far a useful background for studies aimed to decipher the mechanisms that in fish provide adaptive flexibility to the heart in response to environmental stress. They may also be useful to complement results deriving from more traditional models, in order to better understand the response of the more fragile mammalian heart to oxygen levels variations.

## Data Availability

No new data were created or analyzed in this study. Data sharing is not applicable to this article.

## References

[1] Chapman L.J., Mckenzie D.J. (2009). Behavioral responses and ecological consequences. Fish Physiology.

[2] Urbina M.A., Forster M.E., Glover C.N. (2011). Leap of faith: Voluntary emersion behaviour and physiological adaptations to aerial exposure in a non-aestivating freshwater fish in response to aquatic hypoxia. Physiol. Behav..

[3] Richards J.G., Jeffrey G., Richards A.P.F., Colin J.B. (2009). Metabolic and molecular responses of fish to hypoxia. Fish Physiology.

[4] Rogers N.J., Urbina M.A., Reardon E.E., McKenzie D.J., Wilson R.W. (2016). A new analysis of hypoxia tolerance in fishes using a database of critical oxygen level (P crit). Conserv. Physiol..

[5] Ultsch G.R., Regan M.D. (2019). The utility and determination of P(crit) in fishes. J. Exp. Biol..

[6] Farrell A.P., Richards J.G. (2009). Defining hypoxia: An integrative synthesis of the responses of fish to hypoxia. Fish Physiology.

[7] Richards J.G., Arturo Navas C., Carvalho J.E. (2010). Metabolic Rate Suppression as a Mechanism for Surviving Environmental Challenge in Fish. Aestivation: Molecular and Physiological Aspects.

[8] Perry S., Jonz M., Gilmour K. (2009). Oxygen sensing and the hypoxic ventilatory response. Fish Physiology.

[9] Porteus C., Hedrick M.S., Hicks J.W., Wang T., Milsom W.K. (2011). Time domains of the hypoxic ventilatory response in ectothermic vertebrates. J. Comp. Physiol. B.

[10] Reid S.G., Bernier N.J., Perry S.F. (1998). The adrenergic stress response in fish: Control of catecholamine storage and release. Comp. Biochem. Physiol. Part C Pharmacol. Toxicol. Endocrinol..

[11] Farrell A. (2007). Tribute to PL Lutz: A message from the heart–why hypoxic bradycardia in fishes?. J. Exp. Biol..

[12] Fritsche R., Nilsson S. (1993). Cardiovascular and ventilatory control during hypoxia. Fish Ecophysiol..

[13] Harwood C.L., Howarth F.C., Altringham J.D., White E. (2000). Rate-dependent changes in cell shortening, intracellular Ca (2+) levels and membrane potential in single, isolated rainbow trout (*Oncorhynchus mykiss*) ventricular myocytes. J. Exp. Biol..

[14] Brette F., Luxan G., Cros C., Dixey H., Wilson C., Shiels H.A. (2008). Characterization of isolated ventricular myocytes from adult zebrafish (*Danio rerio*). Biochem. Biophys. Res. Commun..

[15] Shiels H.A., Vornanen M., Farrell A.P. (2002). The force–frequency relationship in fish hearts—A review. Comp. Biochem. Physiol. Part A Mol. Integr. Physiol..

[16] Gamperl A., Pinder A., Grant R., Boutilier R. (1994). Influence of hypoxia and adrenaline administration on coronary blood flow and cardiac performance in seawater rainbow trout (*Oncorhynchus mykiss*). J. Exp. Biol..

[17] Barrionuevo W., Fernandes M., Rocha O. (2010). Aerobic and anaerobic metabolism for the zebrafish, Danio rerio, reared under normoxic and hypoxic conditions and exposed to acute hypoxia during development. Braz. J. Biol..

[18] Barrionuevo W., Burggren W. (1999). O_2_ consumption and heart rate in developing zebrafish (*Danio rerio*): Influence of temperature and ambient O_2_. Am. J. Physiol.-Regul. Integr. Comp. Physiol..

[19] Tzaneva V., Perry S.F. (2016). Evidence for a role of heme oxygenase-1 in the control of cardiac function in zebrafish (*Danio rerio*) larvae exposed to hypoxia. J. Exp. Biol..

[20] Schwerte T., Prem C., Mairösl A., Pelster B. (2006). Development of the sympatho-vagal balance in the cardiovascular system in zebrafish (*Danio rerio*) characterized by power spectrum and classical signal analysis. J. Exp. Biol..

[21] Steele S.L., Yang X., Debiais-Thibaud M., Schwerte T., Pelster B., Ekker M., Tiberi M., Perry S.F. (2011). In vivo and in vitro assessment of cardiac β-adrenergic receptors in larval zebrafish (*Danio rerio*). J. Exp. Biol..

[22] Miller S.C., Gillis T.E., Wright P.A. (2011). The ontogeny of regulatory control of the rainbow trout (*Oncorhynchus mykiss*) heart and how this is influenced by chronic hypoxia exposure. J. Exp. Biol..

[23] Joyce W., Wang T. (2022). Regulation of heart rate in vertebrates during hypoxia: A comparative overview. Acta Physiol..

[24] Axelsson M., Farrell A.P., Nilsson S. (1990). Effects of hypoxia and drugs on the cardiovascular dynamics of the Atlantic hagfish Myxine glutinosa. J. Exp. Biol..

[25] Petersen L., Gamperl A. (2010). In situ cardiac function in Atlantic cod (*Gadus morhua*): Effects of acute and chronic hypoxia. J. Exp. Biol..

[26] Farrell A.P., Jones D., Hoar W., Randall D. (1992). The heart. Cardiovasc. Syst..

[27] Imbrogno S., Filice M., Cerra M.C. (2019). Exploring cardiac plasticity in teleost: The role of humoral modulation. Gen. Comp. Endocrinol..

[28] Stecyk J.A., Stenslokken K.O., Farrell A.P., Nilsson G.E. (2004). Maintained cardiac pumping in anoxic crucian carp. Science.

[29] Farrell A.P., Stecyk J.A. (2007). The heart as a working model to explore themes and strategies for anoxic survival in ectothermic vertebrates. Comp. Biochem. Physiol. Part A Mol. Integr. Physiol..

[30] Speers-Roesch B., Sandblom E., Lau G.Y., Farrell A.P., Richards J.G. (2010). Effects of environmental hypoxia on cardiac energy metabolism and performance in tilapia. Am. J. Physiol.-Regul. Integr. Comp. Physiol..

[31] Imbrogno S., Capria C., Tota B., Jensen F.B. (2014). Nitric oxide improves the hemodynamic performance of the hypoxic goldfish (*Carassius auratus*) heart. Nitric Oxide.

[32] Petersen L., Gamperl A. (2010). Effect of acute and chronic hypoxia on the swimming performance, metabolic capacity and cardiac function of Atlantic cod (*Gadus morhua*). J. Exp. Biol..

[33] Motyka R., Norin T., Petersen L.H., Huggett D.B., Gamperl A.K. (2017). Long-term hypoxia exposure alters the cardiorespiratory physiology of steelhead trout (*Oncorhynchus mykiss*), but does not affect their upper thermal tolerance. J. Therm. Biol..

[34] Carnevale C., Roberts J.C., Syme D.A., Gamperl A.K. (2020). Hypoxic acclimation negatively impacts the contractility of steelhead trout (*Oncorhynchus mykiss*) spongy myocardium. Am. J. Physiol.-Regul. Integr. Comp. Physiol..

[35] Burleson M.L., Silva P.E. (2011). Cross tolerance to environmental stressors: Effects of hypoxic acclimation on cardiovascular responses of channel catfish (*Ictalurus punctatus*) to a thermal challenge. J. Therm. Biol..

[36] Burleson M.L., Carlton A.L., Silva P.E. (2002). Cardioventilatory effects of acclimatization to aquatic hypoxia in channel catfish. Respir. Physiol. Neurobiol..

[37] Blažka P. (1958). The Anaerobic Metabolism of Fish. Physiol. Zool..

[38] Piironen J., Holopainen I.J. (1986). A note on seasonality in anoxia tolerance of crucian carp (*Carassius carassius* (L.)) in the laboratory. Ann. Zool. Fenn..

[39] Tikkanen E., Haverinen J., Egginton S., Hassinen M., Vornanen M. (2017). Effects of prolonged anoxia on electrical activity of the heart in crucian carp (*Carassius carassius*). J. Exp. Biol..

[40] Nichols C.G., Ripoll C., Lederer W.J. (1991). ATP-sensitive potassium channel modulation of the guinea pig ventricular action potential and contraction. Circ. Res..

[41] Paajanen V., Vornanen M. (2003). Effects of Chronic Hypoxia on Inward Rectifier K+Current (IK1) in Ventricular Myocytes of Crucian Carp (*Carassiuscarassius*) Heart. J. Membr. Biol..

[42] Chen J., Zhu J.X., Wilson I., Cameron J.S. (2005). Cardioprotective effects of KATP channel activation during hypoxia in goldfish Carassius auratus. J. Exp. Biol..

[43] Hochachka P.W. (1986). Defense strategies against hypoxia and hypothermia. Science.

[44] Bickler P.E., Buck L.T. (2007). Hypoxia tolerance in reptiles, amphibians, and fishes: Life with variable oxygen availability. Annu. Rev. Physiol..

[45] Shoubridge E.A., Hochachka P.W. (1980). Ethanol: Novel end product of vertebrate anaerobic metabolism. Science.

[46] Fagernes C.E., Stenslokken K.O., Rohr A.K., Berenbrink M., Ellefsen S., Nilsson G.E. (2017). Extreme anoxia tolerance in crucian carp and goldfish through neofunctionalization of duplicated genes creating a new ethanol-producing pyruvate decarboxylase pathway. Sci. Rep..

[47] Gattuso A., Garofalo F., Cerra M.C., Imbrogno S. (2018). Hypoxia Tolerance in Teleosts: Implications of Cardiac Nitrosative Signals. Front. Physiol..

[48] Imbrogno S., Aiello D., Filice M., Leo S., Mazza R., Cerra M.C., Napoli A. (2019). MS-based proteomic analysis of cardiac response to hypoxia in the goldfish (*Carassius auratus*). Sci. Rep..

[49] Penhoet E.E., Kochman M., Rutter W.J. (1969). Molecular and catalytic properties of aldolase C. Biochemistry.

[50] Penhoet E.E., Rutter W.J. (1971). Catalytic and immunochemical properties of homomeric and heteromeric combinations of aldolase subunits. J. Biol. Chem..

[51] Brooks S.P.J., Storey K.B., Hochachka P.W., Mommsen T.P. (1995). Is glycolytic rate controlledby the reversible binding of enzymes to subcellular structures?. Biochemistry and Molecular Biology of Fishes.

[52] Treberg J.R., MacCormack T.J., Lewis J.M., Almeida-Val V.M., Val A.L., Driedzic W.R. (2007). Intracellular glucose and binding of hexokinase and phosphofructokinase to particulate fractions increase under hypoxia in heart of the amazonian armored catfish (*Liposarcus pardalis*). Physiol. Biochem. Zool..

[53] Duncan J.A., Storey K.B. (1991). Role of enzyme binding in muscle metabolism of the goldfish. Can. J. Zool..

[54] Sidell B.D., Stowe D.B., Hansen C.A. (1984). Carbohydrate Is the Preferred Metabolic Fuel of the Hagfish (*Myxine glutinosa*) Heart. Physiol. Zool..

[55] Imbrogno S. (2013). The eel heart: Multilevel insights into functional organ plasticity. J. Exp. Biol..

[56] Bailey J.R., MacDougall R., Clowe S., Driedzic W.R. (2000). Anoxic performance of the american eel (*Anguilla rostrata* L.) heart requires extracellular glucose. J. Exp. Zool..

[57] Vornanen M., Haverinen J. (2016). Glycogen dynamics of crucian carp (*Carassius carassius*) in prolonged anoxia. J. Comp. Physiol. B Biochem. Syst. Environ. Physiol..

[58] Shoubridge E.A., Hochachka P.W. (1983). The integrationand control of metabolism in the anoxic goldfish. Mol. Physiol..

[59] Farhat E., Turenne E.D., Choi K., Weber J.M. (2019). Hypoxia-induced remodelling of goldfish membranes. Comp. Biochem. Physiol. Part B Biochem. Mol. Biol..

[60] Farhat E., Cheng H., Romestaing C., Pamenter M., Weber J.M. (2021). Goldfish Response to Chronic Hypoxia: Mitochondrial Respiration, Fuel Preference and Energy Metabolism. Metabolites.

[61] Pedersen C.L., Faggiano S., Helbo S., Gesser H., Fago A. (2010). Roles of nitric oxide, nitrite and myoglobin on myocardial efficiency in trout (*Oncorhynchus mykiss*) and goldfish (*Carassius auratus*): Implications for hypoxia tolerance. J. Exp. Biol..

[62] Pamenter M.E. (2014). Mitochondria: A multimodal hub of hypoxia tolerance. Can. J. Zool..

[63] Du S.N.N., Mahalingam S., Borowiec B.G., Scott G.R. (2016). Mitochondrial physiology and reactive oxygen species production are altered by hypoxia acclimation in killifish (*Fundulus heteroclitus*). J. Exp. Biol..

[64] Onukwufor J.O., Stevens D., Kamunde C. (2017). Combined effects of cadmium, temperature and hypoxia-reoxygenation on mitochondrial function in rainbow trout (*Oncorhynchus mykiss*). Aquat. Toxicol..

[65] Sappal R., MacDougald M., Fast M., Stevens D., Kibenge F., Siah A., Kamunde C. (2015). Alterations in mitochondrial electron transport system activity in response to warm acclimation, hypoxia-reoxygenation and copper in rainbow trout, Oncorhynchus mykiss. Aquat. Toxicol..

[66] Cook D.G., Iftikar F.I., Baker D.W., Hickey A.J.R., Herbert N.A. (2013). Low-O_2_ acclimation shifts the hypoxia avoidance behaviour of snapper (*Pagrus auratus*) with only subtle changes in aerobic and anaerobic function. J. Exp. Biol..

[67] Hickey A.J.R., Renshaw G.M.C., Speers-Roesch B., Richards J.G., Wang Y., Farrell A.P., Brauner C.J. (2012). A radical approach to beating hypoxia: Depressed free radical release from heart fibres of the hypoxia-tolerant epaulette shark (*Hemiscyllum ocellatum*). J. Comp. Physiol. B.

[68] Gerber L., Clow K.A., Katan T., Emam M., Leeuwis R.H.J., Parrish C.C., Gamperl A.K. (2019). Cardiac mitochondrial function, nitric oxide sensitivity and lipid composition following hypoxia acclimation in sablefish. J. Exp. Biol..

[69] St-Pierre J., Boutilier R.G. (2001). Aerobic capacity of frog skeletal muscle during hibernation. Physiol. Biochem. Zool..

[70] O’Brien K.M. (2011). Mitochondrial biogenesis in cold-bodied fishes. J. Exp. Biol..

[71] Filice M., Barca A., Amelio D., Leo S., Mazzei A., Del Vecchio G., Verri T., Cerra M.C., Imbrogno S. (2021). Morpho-functional remodelling of the adult zebrafish (*Danio rerio*) heart in response to waterborne angiotensin II exposure. Gen. Comp. Endocrinol..

[72] Cerra M.C., Imbrogno S., Amelio D., Garofalo F., Colvee E., Tota B., Icardo J.M. (2004). Cardiac morphodynamic remodelling in the growing eel (*Anguilla anguilla* L.). J. Exp. Biol..

[73] Farhat E., Talarico G.G.M., Grégoire M., Weber J.M., Mennigen J.A. (2022). Epigenetic and post-transcriptional repression support metabolic suppression in chronically hypoxic goldfish. Sci. Rep..

[74] Fago A., Jensen F.B. (2015). Hypoxia tolerance, nitric oxide, and nitrite: Lessons from extreme animals. Physiology.

[75] Imbrogno S., Verri T., Filice M., Barca A., Schiavone R., Gattuso A., Cerra M.C. (2022). Shaping the cardiac response to hypoxia: NO and its partners in teleost fish. Curr. Res. Physiol..

[76] Filice M., Mazza R., Leo S., Gattuso A., Cerra M.C., Imbrogno S. (2020). The Hypoxia Tolerance of the Goldfish (Carassius auratus) Heart: The NOS/NO System and Beyond. Antioxidants.

[77] Filice M., Imbrogno S., Gattuso A., Cerra M.C. (2021). Hypoxic and Thermal Stress: Many Ways Leading to the NOS/NO System in the Fish Heart. Antioxidants.

[78] Filice M., Cerra M.C., Imbrogno S. (2022). The goldfish Carassius auratus: An emerging animal model for comparative cardiac research. J. Comp. Physiol. B Biochem. Syst. Environ. Physiol..

[79] Imbrogno S., Tota B., Gattuso A. (2011). The evolutionary functions of cardiac NOS/NO in vertebrates tracked by fish and amphibian paradigms. Nitric Oxide.

[80] Imbrogno S., Filice M., Cerra M.C., Gattuso A. (2018). NO, CO and H2 S: What about gasotransmitters in fish and amphibian heart?. Acta Physiol..

[81] Garofalo F., Imbrogno S., Tota B., Amelio D. (2012). Morpho-functional characterization of the goldfish (*Carassius auratus* L.) heart. Comp. Biochem. Physiol. Part A Mol. Integr. Physiol..

[82] Imbrogno S., Garofalo F., Cerra M.C., Mahata S.K., Tota B. (2010). The catecholamine release-inhibitory peptide catestatin (chromogranin A344-363) modulates myocardial function in fish. J. Exp. Biol..

[83] Filice M., Amelio D., Garofalo F., David S., Fucarino A., Jensen F.B., Imbrogno S., Cerra M.C. (2017). Angiotensin II dependent cardiac remodeling in the eel Anguilla anguilla involves the NOS/NO system. Nitric Oxide.

[84] Tota B., Amelio D., Cerra M.C., Garofalo F. (2018). The morphological and functional significance of the NOS/NO system in the respiratory, osmoregulatory, and contractile organs of the African lungfish. Acta Histochem..

[85] Andreakis N., D’Aniello S., Albalat R., Patti F.P., Garcia-Fernàndez J., Procaccini G., Sordino P., Palumbo A. (2011). Evolution of the nitric oxide synthase family in metazoans. Mol. Biol. Evol..

[86] Hetrick E.M., Schoenfisch M.H. (2009). Analytical chemistry of nitric oxide. Annu. Rev. Anal. Chem..

[87] Lundberg J.O., Weitzberg E., Gladwin M.T. (2008). The nitrate-nitrite-nitric oxide pathway in physiology and therapeutics. Nat. Rev. Drug Discov..

[88] van Faassen E.E., Bahrami S., Feelisch M., Hogg N., Kelm M., Kim-Shapiro D.B., Kozlov A.V., Li H., Lundberg J.O., Mason R. (2009). Nitrite as regulator of hypoxic signaling in mammalian physiology. Med. Res. Rev..

[89] Zweier J.L., Samouilov A., Kuppusamy P. (1999). Non-enzymatic nitric oxide synthesis in biological systems. Biochim. Biophys. Acta.

[90] Angelone T., Gattuso A., Imbrogno S., Mazza R., Tota B. (2012). Nitrite is a positive modulator of the Frank-Starling response in the vertebrate heart. Am. J. Physiol. Regul. Integr. Comp. Physiol..

[91] Omar S.A., Webb A.J. (2014). Nitrite reduction and cardiovascular protection. J. Mol. Cell. Cardiol..

[92] Giordano D., Pesce A., Vermeylen S., Abbruzzetti S., Nardini M., Marchesani F., Berghmans H., Seira C., Bruno S., Javier Luque F. (2020). Structural and functional properties of Antarctic fish cytoglobins-1: Cold-reactivity in multi-ligand reactions. Comput. Struct. Biotechnol. J..

[93] Corti P., Xue J., Tejero J., Wajih N., Sun M., Stolz D.B., Tsang M., Kim-Shapiro D.B., Gladwin M.T. (2016). Globin X is a six-coordinate globin that reduces nitrite to nitric oxide in fish red blood cells. Proc. Natl. Acad. Sci. USA.

[94] Hansen M.N., Lundberg J.O., Filice M., Fago A., Christensen N.M., Jensen F.B. (2016). The roles of tissue nitrate reductase activity and myoglobin in securing nitric oxide availability in deeply hypoxic crucian carp. J. Exp. Biol..

[95] Jansson E.A., Huang L., Malkey R., Govoni M., Nihlén C., Olsson A., Stensdotter M., Petersson J., Holm L., Weitzberg E. (2008). A mammalian functional nitrate reductase that regulates nitrite and nitric oxide homeostasis. Nat. Chem. Biol..

[96] Gladwin M.T., Kim-Shapiro D.B. (2008). The functional nitrite reductase activity of the heme-globins. Blood J. Am. Soc. Hematol..

[97] Tejero J., Gladwin M.T. (2014). The globin superfamily: Functions in nitric oxide formation and decay. Biol. Chem..

[98] Li H., Samouilov A., Liu X., Zweier J.L. (2001). Characterization of the magnitude and kinetics of xanthine oxidase-catalyzed nitrite reduction. Evaluation of its role in nitric oxide generation in anoxic tissues. J. Biol. Chem..

[99] Hansen M.N., Gerber L., Jensen F.B. (2016). Nitric oxide availability in deeply hypoxic crucian carp: Acute and chronic changes and utilization of ambient nitrite reservoirs. Am. J. Physiol. Regul. Integr. Comp. Physiol..

[100] Hansen M.N., Jensen F.B. (2010). Nitric oxide metabolites in goldfish under normoxic and hypoxic conditions. J. Exp. Biol..

[101] Jensen F.B. (2009). The role of nitrite in nitric oxide homeostasis: A comparative perspective. Biochim. Biophys. Acta.

[102] Sandvik G.K., Nilsson G.E., Jensen F.B. (2012). Dramatic increase of nitrite levels in hearts of anoxia-exposed crucian carp supporting a role in cardioprotection. Am. J. Physiol. Regul. Integr. Comp. Physiol..

[103] Jensen F.B. (2007). Nitric oxide formation from nitrite in zebrafish. J. Exp. Biol..

[104] Jensen F.B., Hansen M.N. (2011). Differential uptake and metabolism of nitrite in normoxic and hypoxic goldfish. Aquat. Toxicol..

[105] Grisham M.B., Jourd’Heuil D., Wink D.A. (1999). Nitric oxide. I. Physiological chemistry of nitric oxide and its metabolites: Implications in inflammation. Am. J. Physiol..

[106] Montfort W.R., Wales J.A., Weichsel A. (2017). Structure and Activation of Soluble Guanylyl Cyclase, the Nitric Oxide Sensor. Antioxid. Redox Signal..

[107] Poulos T.L. (2006). Soluble guanylate cyclase. Curr. Opin. Struct. Biol..

[108] Padayatti P.S., Pattanaik P., Ma X., van den Akker F. (2004). Structural insights into the regulation and the activation mechanism of mammalian guanylyl cyclases. Pharmacol. Ther..

[109] Derbyshire E.R., Marletta M.A. (2012). Structure and regulation of soluble guanylate cyclase. Annu. Rev. Biochem..

[110] Rybalkin S.D., Yan C., Bornfeldt K.E., Beavo J.A. (2003). Cyclic GMP phosphodiesterases and regulation of smooth muscle function. Circ. Res..

[111] Ignarro L.J., Buga G.M., Wood K.S., Byrns R.E., Chaudhuri G. (1987). Endothelium-derived relaxing factor produced and released from artery and vein is nitric oxide. Proc. Natl. Acad. Sci. USA.

[112] Radi R. (2004). Nitric oxide, oxidants, and protein tyrosine nitration. Proc. Natl. Acad. Sci. USA.

[113] Douki T., Cadet J. (1996). Peroxynitrite mediated oxidation of purine bases of nucleosides and isolated DNA. Free. Radic. Res..

[114] Martemucci G., Costagliola C., Mariano M., D’andrea L., Napolitano P., D’Alessandro A.G. (2022). Free Radical Properties, Source and Targets, Antioxidant Consumption and Health. Oxygen.

[115] Tota B., Imbrogno S., Mannarino C., Mazza R. (2004). Vasostatins and Negative Inotropy in Vertebrate Hearts. Curr. Med. Chem. -Immunol. Endocr. Metab. Agents.

[116] Amelio D., Garofalo F., Capria C., Tota B., Imbrogno S. (2013). Effects of temperature on the nitric oxide-dependent modulation of the Frank-Starling mechanism: The fish heart as a case study. Comp. Biochem. Physiol. Part A Mol. Integr. Physiol..

[117] Imbrogno S., Cerra M.C., Gamperl A.K., Gillis T.E., Farrell A.P., Brauner C.J. (2017). 5—Hormonal and Autacoid Control of Cardiac Function. Fish Physiology.

[118] Filice M., Mazza R., Imbrogno S., Mileti O., Baldino N., Barca A., Del Vecchio G., Verri T., Gattuso A., Cerra M.C. (2022). An ACE2-Alamandine Axis Modulates the Cardiac Performance of the Goldfish Carassius auratus via the NOS/NO System. Antioxidants.

[119] Eddy F.B., Tibbs P. (2003). Effects of nitric oxide synthase inhibitors and a substrate, l-arginine, on the cardiac function of juvenile salmonid fish. Comp. Biochem. Physiol. Part C Toxicol. Pharmacol..

[120] Sykes B.G., Van Steyn P.M., Vignali J.D., Winalski J., Lozier J., Bell W.E., Turner J.E. (2016). The Relationship between Estrogen and Nitric Oxide in the Prevention of Cardiac and Vascular Anomalies in the Developing Zebrafish (*Danio Rerio*). Brain Sci..

[121] Siamwala J.H., Kumar P., Veeriah V., Muley A., Rajendran S., Konikkat S., Majumder S., Mani K.P., Chatterjee S. (2019). Nitric Oxide Reverses the Position of the Heart during Embryonic Development. Int. J. Mol. Sci..

[122] Kopp R., Bauer I., Ramalingam A., Egg M., Schwerte T. (2014). Prolonged hypoxia increases survival even in Zebrafish (Danio rerio) showing cardiac arrhythmia. PLoS ONE.

[123] Imbrogno S., Angelone T., Adamo C., Pulera E., Tota B., Cerra M.C. (2006). Beta3-adrenoceptor in the eel (Anguilla anguilla) heart: Negative inotropy and NO-cGMP-dependent mechanism. J. Exp. Biol..

[124] Taylor C.T., Moncada S. (2010). Nitric oxide, cytochrome C oxidase, and the cellular response to hypoxia. Arterioscler. Thromb. Vasc. Biol..

[125] Davidson S.M., Duchen M.R. (2006). Effects of NO on mitochondrial function in cardiomyocytes: Pathophysiological relevance. Cardiovasc. Res..

[126] Erusalimsky J.D., Moncada S. (2007). Nitric oxide and mitochondrial signaling: From physiology to pathophysiology. Arterioscler. Thromb. Vasc. Biol..

[127] Korge P., Ping P., Weiss J.N. (2008). Reactive oxygen species production in energized cardiac mitochondria during hypoxia/reoxygenation: Modulation by nitric oxide. Circ. Res..

[128] Palacios-Callender M., Quintero M., Hollis V.S., Springett R.J., Moncada S. (2004). Endogenous NO regulates superoxide production at low oxygen concentrations by modifying the redox state of cytochrome c oxidase. Proc. Natl. Acad. Sci. USA.

[129] Brown G.C. (2001). Regulation of mitochondrial respiration by nitric oxide inhibition of cytochrome c oxidase. Biochim. Biophys. Acta.

[130] Cleeter M.W., Cooper J.M., Darley-Usmar V.M., Moncada S., Schapira A.H. (1994). Reversible inhibition of cytochrome c oxidase, the terminal enzyme of the mitochondrial respiratory chain, by nitric oxide. Implications for neurodegenerative diseases. FEBS Lett..

[131] Cooper C.E., Mason M.G., Nicholls P. (2008). A dynamic model of nitric oxide inhibition of mitochondrial cytochrome c oxidase. Biochim. Biophys. Acta.

[132] Brown G.C., Borutaite V. (2004). Inhibition of mitochondrial respiratory complex I by nitric oxide, peroxynitrite and S-nitrosothiols. Biochim. Biophys. Acta.

[133] Chouchani E.T., Methner C., Nadtochiy S.M., Logan A., Pell V.R., Ding S., James A.M., Cochemé H.M., Reinhold J., Lilley K.S. (2013). Cardioprotection by S-nitrosation of a cysteine switch on mitochondrial complex I. Nat. Med..

[134] Clementi E., Brown G.C., Feelisch M., Moncada S. (1998). Persistent inhibition of cell respiration by nitric oxide: Crucial role of S-nitrosylation of mitochondrial complex I and protective action of glutathione. Proc. Natl. Acad. Sci. USA.

[135] Cameron J.S., Hoffmann K.E., Zia C., Hemmett H.M., Kronsteiner A., Lee C.M. (2003). A role for nitric oxide in hypoxia-induced activation of cardiac KATP channels in goldfish (*Carassius auratus*). J. Exp. Biol..

[136] Noma A. (1983). ATP-regulated K^+^ channels in cardiac muscle. Nature.

[137] Angelone T., Quintieri A.M., Pasqua T., Filice E., Cantafio P., Scavello F., Rocca C., Mahata S.K., Gattuso A., Cerra M.C. (2015). The NO stimulator, Catestatin, improves the Frank-Starling response in normotensive and hypertensive rat hearts. Nitric Oxide.

[138] Strijdom H., Friedrich S.O., Hattingh S., Chamane N., Lochner A. (2009). Hypoxia-induced regulation of nitric oxide synthase in cardiac endothelial cells and myocytes and the role of the PI3-K/PKB pathway. Mol. Cell. Biochem..

[139] Rahman M.S., Thomas P. (2007). Molecular cloning, characterization and expression of two hypoxia-inducible factor alpha subunits, HIF-1alpha and HIF-2alpha, in a hypoxia-tolerant marine teleost, Atlantic croaker (*Micropogonias undulatus*). Gene.

[140] O’Brien K.M., Rix A.S., Grove T.J., Sarrimanolis J., Brooking A., Roberts M., Crockett E.L. (2020). Characterization of the hypoxia-inducible factor-1 pathway in hearts of Antarctic notothenioid fishes. Comp. Biochem. Physiol. Part B Biochem. Mol. Biol..

[141] Mandic M., Joyce W., Perry S.F. (2021). The evolutionary and physiological significance of the Hif pathway in teleost fishes. J. Exp. Biol..

[142] Jugdutt B.I. (2002). Nitric oxide and cardioprotection during ischemia-reperfusion. Heart Fail. Rev..

[143] Hochachka P.W., Lutz P.L. (2001). Mechanism, origin, and evolution of anoxia tolerance in animals. Comp. Biochem. Physiol. Part B Biochem. Mol. Biol..

[144] Liu L., Simon M.C. (2004). Regulation of transcription and translation by hypoxia. Cancer Biol. Ther..

[145] Semenza G.L. (2007). Hypoxia-inducible factor 1 (HIF-1) pathway. Sci. STKE Signal Transduct. Knowl. Environ..

[146] Mateo J., García-Lecea M., Cadenas S., Hernández C., Moncada S. (2003). Regulation of hypoxia-inducible factor-1alpha by nitric oxide through mitochondria-dependent and -independent pathways. Biochem. J..

[147] Garofalo F., Parisella M.L., Amelio D., Tota B., Imbrogno S. (2009). Phospholamban S-nitrosylation modulates Starling response in fish heart. Proc. Biol. Sci..

[148] Imbrogno S., Mazza R., Pugliese C., Filice M., Angelone T., Loh Y.P., Tota B., Cerra M.C. (2017). The Chromogranin A-derived sympathomimetic serpinin depresses myocardial performance in teleost and amphibian hearts. Gen. Comp. Endocrinol..

[149] Durham W.J., Aracena-Parks P., Long C., Rossi A.E., Goonasekera S.A., Boncompagni S., Galvan D.L., Gilman C.P., Baker M.R., Shirokova N. (2008). RyR1 S-nitrosylation underlies environmental heat stroke and sudden death in Y522S RyR1 knockin mice. Cell.

[150] Sips P.Y., Irie T., Zou L., Shinozaki S., Sakai M., Shimizu N., Nguyen R., Stamler J.S., Chao W., Kaneki M. (2013). Reduction of cardiomyocyte S-nitrosylation by S-nitrosoglutathione reductase protects against sepsis-induced myocardial depression. Am. J. Physiol. Heart Circ. Physiol..

[151] Mazza R., Gattuso A., Imbrogno S., Boukhzar L., Leo S., Mallouki B.Y., Filice M., Rocca C., Angelone T., Anouar Y. (2019). Selenoprotein T as a new positive inotrope in the goldfish, Carassius auratus. J. Exp. Biol..

[152] Ischiropoulos H. (2003). Biological selectivity and functional aspects of protein tyrosine nitration. Biochem. Biophys. Res. Commun..

[153] Bigelow D.J. (2009). Nitrotyrosine-modified SERCA2: A cellular sensor of reactive nitrogen species. Pflug. Arch. Eur. J. Physiol..

[154] Braun J.L., Hamstra S.I., Messner H.N., Fajardo V.A. (2019). SERCA2a tyrosine nitration coincides with impairments in maximal SERCA activity in left ventricles from tafazzin-deficient mice. Physiol. Rep..

[155] Cerra M.C., Imbrogno S. (2012). Phospholamban and cardiac function: A comparative perspective in vertebrates. Acta Physiol..

[156] Cadenas E. (2004). Mitochondrial free radical production and cell signaling. Mol. Asp. Med..

[157] Imbrogno S., Gattuso A., Mazza R., Angelone T., Cerra M.C. (2015). β3 -AR and the vertebrate heart: A comparative view. Acta Physiol..

[158] Korajoki H., Vornanen M. (2012). Expression of SERCA and phospholamban in rainbow trout (*Oncorhynchus mykiss*) heart: Comparison of atrial and ventricular tissue and effects of thermal acclimation. J. Exp. Biol..

[159] Leo S., Gattuso A., Mazza R., Filice M., Cerra M.C., Imbrogno S. (2019). Cardiac influence of the beta3-adrenoceptor in the goldfish (*Carassius auratus*): A protective role under hypoxia?. J. Exp. Biol..

[160] Balligand J.L. (2016). Cardiac salvage by tweaking with beta-3-adrenergic receptors. Cardiovasc. Res..

[161] Dal Monte M., Filippi L., Bagnoli P. (2013). Beta3-adrenergic receptors modulate vascular endothelial growth factor release in response to hypoxia through the nitric oxide pathway in mouse retinal explants. Naunyn-Schmiedeberg’s Arch. Pharmacol..

